# Effects of *ADAMTS14* genetic polymorphism and cigarette smoking on the clinicopathologic development of hepatocellular carcinoma

**DOI:** 10.1371/journal.pone.0172506

**Published:** 2017-02-23

**Authors:** Ming-Jen Sheu, Ming-Ju Hsieh, Ying-Erh Chou, Po-Hui Wang, Chao-Bin Yeh, Shun-Fa Yang, Hsiang-Lin Lee, Yu-Fan Liu

**Affiliations:** 1 Department of Gastroenterology and Hepatology, Chi Mei Medical Center, Tainan, Taiwan; 2 Institute of Medicine, Chung Shan Medical University, Taichung, Taiwan; 3 Cancer Research Center, Changhua Christian Hospital, Changhua, Taiwan; 4 Graduate Institute of Biomedical Sciences, China Medical University, Taichung, Taiwan; 5 School of Medicine, Chung Shan Medical University, Taichung, Taiwan; 6 Department of Medical Research, Chung Shan Medical University Hospital, Taichung, Taiwan; 7 Department of Obstetrics and Gynecology, Chung Shan Medical University Hospital, Taichung, Taiwan; 8 Department of Emergency Medicine, Chung Shan Medical University Hospital, Taichung, Taiwan; 9 Deptartment of Surgery, Chung Shan Medical University Hospital, Taichung, Taiwan; 10 Department of Biomedical Sciences, College of Medicine Sciences and Technology, Chung Shan Medical University, Taichung, Taiwan; 11 Division of Allergy, Department of Pediatrics, Chung-Shan Medical University Hospital, Taichung, Taiwan; Taipei Medical University, TAIWAN

## Abstract

**Background:**

*ADAMTS14* is a member of the ADAMTS (a disintegrin and metalloproteinase with thrombospondin motifs), which are proteolytic enzymes with a variety of further ancillary domain in the C-terminal region for substrate specificity and enzyme localization via extracellular matrix association. However, whether *ADAMTS14* genetic variants play a role in hepatocellular carcinoma (HCC) susceptibility remains unknown.

**Methodology/Principal findings:**

Four non-synonymous single-nucleotide polymorphisms (nsSNPs) of the *ADAMTS14* gene were examined from 680 controls and 340 patients with HCC. Among 141 HCC patients with smoking behaviour, we found significant associations of the rs12774070 (CC+AA vs CC) and rs61573157 (CT+TT vs CC) variants with a clinical stage of HCC (OR: 2.500 and 2.767; 95% CI: 1.148–5.446 and 1.096–6.483; *P* = 0.019 and 0.026, respectively) and tumour size (OR: 2.387 and 2.659; 95% CI: 1.098–5.188 and 1.055–6.704; *P* = 0.026 and 0.034, respectively), but not with lymph node metastasis or other clinical statuses. Moreover, an additional integrated *in silico* analysis proposed that rs12774070 and rs61573157 affected essential post-translation *O*-glycosylation site within the 3^rd^ thrombospondin type 1 repeat and a novel proline-rich region embedded within the C-terminal extension, respectively.

**Conclusions:**

Taken together, our results suggest an involvement of *ADAMTS14* SNP rs12774070 and rs61573157 in the liver tumorigenesis and implicate the *ADAMTS14* gene polymorphism as a predict factor during the progression of HCC.

## Introduction

Hepatocellular carcinoma (HCC) is one of the most frequent malignancies of the liver and represents the second and sixth leading cause of cancer related death among males and females in 2012, respectively [1]. HCC carcinogenesis is a multistep and complex etiologic process [2], well-documented environmental and potentially preventable risk factors include major etiologic agents such as chronic hepatitis B virus (HBV) and hepatitis C virus (HCV) infection [3]. HBV accounts for at least 60% of HCC among Asian patients. Currently, the incidence of HCC shows that genetic mutations and environmental factors may increase the severity of the hepatic inflammation that lead to cell differentiation, proliferation and the deposition of connective tissue, which are necessary for the pathophysiology of hepatocarcinogenesis [4–6].

*ADAMTS14* [7–9], which is located on chromosome 10q22.1, belongs to the ADAMTS (also known as a disintegrin and metalloproteinase domain with thrombospondin motifs) zinc ion-dependent proteinase family [10, 11] that contains an N-terminal catalytic domain and a C-terminal ancillary domain which defines substrate specificity including thrombospondin type 1 repeats (TSR) domain [12] and an unique proline-rich region (PRR) [13, 14] that was found to be essential for recognition of its procollagen substrates. The complete human ADAMTS is a name given to a family of 19 secreted, multi-domain proteolytic enzymes, and many of them appear to binds directly to extracellular matrix (ECMs) components, suggesting that the interaction with glycoconjugates through their TSR domain or their other region is responsible for the physiological processes of tumour-induced angiogenesis, invasion and organgenesis [15].

The TSR domain consists of approximately 60 amino acids, has a divalent cation-dependent modular structure, and has been reported to be a critical mediator of cell-matrix interactions. The majority of TSR domains contain six conserved cysteine residues as a long right-handed spiralling antiparallel β sheet and a groove-like structure that might be the “recognition” face, which mediate interactions with ECM components; the domains have been postulated to have roles involved in anti-angiogenesis activity [16]. Furthermore, a novel PRR domain embedded within the C-terminal extension stabilizes the molecule due to the rigidity conferred by its pyrrolidine ring, which leads to steric hindrance in peptidic backbones [13]. The PRR forms a left-handed extended helix of 3.0 residues per turn. PRRs play a major role through intramolecular interactions with other elements in the molecule, and they serve this structure as recognition sites with PRR-recognizing domains, such as Src-homology 3 (SH3) [17] and WW [18] (named after two highly conserved tryptophan residues in the domain) domains.

Although the development of HCC may take 20 to 50 years, early detection of this cancer is seldom accomplished due to the lack of reliable markers. Excessive angiogenesis has been demonstrated to result in a poor diagnosis for HCC, as well as being essential for development of a solid tumour in size. *ADAMTS14* markedly up-regulated expression in human mammary tumours and non-neoplastic breast tissue [19]. In addition, the genetic association of non-synonymous single nucleotide polymorphisms (nsSNPs) in *ADAMTS14* proteases to influence osteoarthritis phenotypes and tumour microenvironment has been reported [20, 21]. To understand the roles in tumour pathobiology [22], here, we conducted a hypothesis-driven case-control study to identify four nsSNPs (rs10823607, rs12774070, rs4747096, and rs61537157; Table 1) in ADAMTS14 that are damaging for protein function, and these were studied with the environmental carcinogens to evaluate HCC susceptibility.

**Table 1:** Variants, position, function, amino acid and changes of observed *ADAMTS14* sequence.

Chromosome #	Exon (contiguous position)			
	10:70,741,007	10:70,753,879	10:70,758,253	10:70,760,503
cDNA position & nucleotide change ^‡^	c.1769T>C	c.2809C>A	c.3146A>G	c.3322C>T
Protein position †	590	936	1,049	1,108
dbSNP (rs number)	rs10823607	rs12774070	rs4747096	rs61573157
Function	Non-synonymous	Non-synonymous	Non-synonymous	Non-synonymous
dbSNP allele	CTG>CGG	CTG>ATG	GAA>GGA	CCA>TCA
Protein residue	Leu>Pro	Leu>Met	Glu>Gly	Pro>Ser

^#^ GRCh37.p13

^†^ NM_080722.4

^‡^ NP_542453.2

## Materials and methods

### Description of the enrolled participants

This hospital-based case control study recruited 340 (249 men and 91 women; mean age = 63.20 ± 11.68 years) HCC patients between 2007 and 2014 at the Chung Shan Medical University Hospital, Taiwan. During the same study period, 680 ethnic group-matched individuals (498 men and 182 women; mean age = 62.43±4.14 years) were enrolled as the controls; these subjects received a physical examination at the same hospital. The TNM classification of the American Joint Committee on Cancer (AJCC) was used for staging of hepatocellular carcinoma [23, 24]. Vascular invasion was defined by the presence of adjacent thrombus to the tumor in portal vein. The Child-Pugh grade is used to assess the prognosis of chronic liver disease. Before commencing the study, approval was obtained from the Institutional Review Board of Chung Shan Medical University Hospital, and informed written consent was obtained from each individual (CSMUH No:CS15099).

### nsSNPs selection and genotyping

Genomic DNA was isolated from peripheral blood using the QIAamp DNA blood mini kit (Qiagen, Valencia, CA, USA) as described in detail previously [25]. DNA was dissolved in TE buffer and used as the template in polymerase chain reactions. Genotyping of four nsSNPs (rs10823607, rs12774070, rs4747096, and rs61537157; Table 1) in *ADAMTS14* with minor allele frequencies >5% in the HapMap Chinese Han Beijing (CHB) population was performed by the TaqMan SNP genotyping assay (Applied Biosystems, Foster City, CA, USA) [26]. Moreover, four selected nsSNPs show alleles of that region with recombination rate plotted and haplotype display in Figure A in S1 File. rs10823607 (529^th^), rs12774070 (621^th^), rs474096 (669^th^), and rs61573157 (689^th^) indicate the independent examined loci that are located on the LD map of haplotype blocks 22, 26, 31, and 32, respectively (Figure A in S1 File).

### Bioinformatics analysis

Several semi-automated bioinformatics tools to assess whether SNPs or their linked genetic variants were associated with a putative function that might affect patient outcomes were utilized [27, 28]. We briefly the *in silico* functional predictions the structural models obtained by *de novo* prediction methods including the architecture of secondary structure arrangements, disorder content, transmembrane helices, coiled-coils and signal peptides using the Ginzu protocol to have demonstrated the utility for determined biological insight, either through protein functional sites recognition or functional annotation by fold identification [29, 30]. In additional, the development of potential active sites and post-translation modification of *N*-glycosylation sites were received from UniProtKB database [31] and determined using SignalP-NN [32]. Moreover, the heterogeneity at amino acid level potential function that the model the primary contribution to structural specificity was determined using software BioEdit Entropy algorithm [33]. The potential interaction of membranes with proteins determined by amphiphilic character obtained the structural information using the 3D hydrophobic moments of helical wheel projections [34] and the Wenxiang diagram coordinate system that represented the disposition of hydrophobic and hydrophilic residues that provided an α-helix can be viewed as a 2D diagram generated by its conical projection (http://www.jci-bioinfo.cn/wenxiang2) [35].”

### Statistical analysis

The Mann-Whitney U test and Fisher’s exact test were used to compare differences in the distribution of age and demographic characteristics between the controls and HCC patients. ORs with 95% confidence intervals (CIs) were estimated using logistic regression models. AORs with 95% CIs were used to assess the association between genotype frequencies with HCC risk and clinical factors. *P* values less than 0.05 were considered significant. The data were analysed with the SPSS 12.0 statistical software (SPSS Inc., Chicago, IL, USA).

## Results

### Study population

A total of 1,020 participants, including 340 HCC cases and 680 controls were successfully genotyped for further analysis. The demographic characteristics including mean age, gender, alcohol consumption, tobacco consumption and disease stage are shown in Table 2. No significant differences between groups existed for age (*P* = 0.124), gender (*P* = 1.00) and tobacco consumption (*P* = 0.442) in the healthy controls and patients with HCC **(**Table 2).

**Table 2:** The distributions of demographical characteristics and clinical parameters in 680 controls and 340 patients with HCC.

Variable	Controls (N = 680)	Patients (N = 340)	*P* value ^†^
**Age (yrs)**	**Mean ± S.D.**	**Mean ± S.D.**	*P* = 0.124
	62.43 ± 4.14	63.20 ± 11.68	
**Gender**	**n (%)**	**n (%)**	
Male	498 (73.2%)	249 (73.2%)	*P* = 1.000
Female	182 (26.8%)	91 (26.8%)	
**Alcohol consumption**			
No	578 (85.0%)	211 (62.1%)	*P*<0.001
Yes	102 (15.0%)	129 (37.9%)	
**Tobacco consumption**			
No	415 (61.0%)	199 (58.5%)	*P* = 0.442
Yes	265 (39.0%)	141 (41.5%)	
**Stage**			
I+II		226 (66.5%)	
III+IV		114 (33.5%)	
**Tumor T status**			
≤ T2		229 (67.4%)	
> T2		111 (32.6%)	
**Lymph node status**			
N0		330 (97.1%)	
N1+N2		10 (2.9%)	
**Metastasis**			
M0		322 (94.7%)	
M1		18 (5.3%)	
**Vascular invasion**			
No		276 (81.2%)	
Yes		64 (18.8%)	

^†^ Mann-Whitney U test or Fisher’s exact test was used between controls and patients with HCC.

### Frequency distribution of ADAMTS14 alleles and their associations with HCC

Table 3 summarized the basic characteristics of *ADAMTS14* nsSNPs in the study population. In these controls, the genotypic frequency of *ADAMTS14* SNP rs10823607 met the Hardy-Weinberg equilibrium (*P* = 0.571, χ^2^ value: 0.32.). The frequencies of *ADAMTS14* SNPs rs12774070, rs4747096, and rs61573157 were also in the Hardy-Weinberg equilibrium (*P* = 0.157, χ^2^ value: 2.00; *P* = 0.528, χ^2^ value: 0.40; and *P* = 0.872, χ^2^ value: 0.03, respectively). According to the adjusted odds ratios (AORs) with their 95% confidence interval (CI) with multiple logistic regression model for HCC of *ADAMTS14* gene polymorphism, we failed to individually detect any significant association of these *ADAMTS14* variants with the occurrence of HCC between the two study groups compared with their corresponding wild-type homozygotes after adjusting for confounding factors (Table 3).

**Table 3:** Distribution frequency of *ADAMTS14* genotypes in 680 controls and 340 patients with HCC.

Variable	Controls (N = 680) n (%)	Patients (N = 340) n (%)	OR (95% CI)	AOR (95% CI)
**rs10823607**				
CC	578 (85.0%)	290 (85.3%)	1.00	1.00
CT	99 (14.6%)	47 (13.8%)	0.946 (0.650–1.376)	0.933 (0.633–1.367)
TT	3 (0.4%)	3 (0.9%)	1.993 (0.400–9.936)	2.249 (0.434–11.661)
CT+TT	102 (15.0%)	50 (14.7%)	0.977 (0.677–1.410)	0.969 (0.663–1.417)
**rs12774070**				
CC	501 (73.7%)	251 (73.8%)	1.00	1.00
CA	160 (23.5%)	82 (24.1%)	1.023 (0.753–1.390)	0.984 (0.716–1.353)
AA	19 (2.8%)	7 (2.1%)	0.735 (0.305–1.772)	0.894 (0.365–2.187)
CA+AA	179 (26.3%)	89 (26.2%)	0.992 (0.738–1.334)	0.976 (0.718–1.326)
**rs4747096**				
AA	270 (39.7%)	139 (40.9%)	1.00	1.00
AG	323 (47.5%)	166 (48.8%)	0.998 (0.757–1.317)	1.010 (0.758–1.345)
GG	87 (12.8%)	35 (10.3%)	0.781 (0.502–1.216)	0.833 (0.528–1.315)
AG+GG	410 (60.3%)	201 (59.1%)	0.952 (0.730–1.242)	0.973 (0.739–1.281)
**rs61573157**				
CC	562 (82.6%)	282 (82.9%)	1.00	1.00
CT	112 (16.5%)	57 (16.8%)	1.014 (0.715–1.439)	0.991 (0.690–1.423)
TT	6 (0.9%)	1 (0.3%)	0.332 (0.040–2.772)	0.453 (0.054–3.790)
CT+TT	118 (17.4%)	58 (17.1%)	0.980 (0.694–1.384)	0.967 (0.677–1.383)

The odds ratios (ORs) and with their 95% confidence intervals (CIs) were estimated by logistic regression models. The adjusted odds ratios (AORs) with their 95% confidence intervals (CIs) were estimated by multiple logistic regression models after controlling for alcohol consumption. * *P* value < 0.05 as statistically significant.

### Effects of interactions between ADAMTS14 variants and smoking behavior in HCC

Tables 4 and 5 show the results of additive and multiple interaction analysis between the two nsSNPs, rs12774070 and rs61573157, with smoking environmental risk factors for HCC. Among 141 HCC patients with smoking behavior in our study, significant associations of rs12774070 (CA+AA vs CC) and rs61573157 (CT+TT vs CC) variants with clinical stage of HCC (OR: 2.500 and 2.767; 95% CI: 1.148–5.446 and 1.096–6.483; *P* = 0.019 and 0.026, respectively) and tumor size (OR: 2.387 and 2.659; 95% CI: 1.098–5.188 and 1.055–6.704; *P* = 0.026 and 0.034, respectively), but not lymph node metastasis and other clinical status, were observed (Tables 4 and 5). Therefore, these polymorphic markers may further improve through the analysis of environmental/behavioral agents and background liver function. However, no significant difference in the serum levels of these biomarkers was detected between patients who possessed at least on polymorphic allele and those who did not have any of the *ADAMTS14* nsSNPs examined. (Table A in S1 File)

**Table 4:** Odds Ratio (OR) and 95% Confidence Interval (CI) of clinical status and *ADAMTS14* rs12774070 genotypic frequencies in 141 HCC patients with tobacco consumption.

Variable	Genotypic frequencies		OR (95% CI)	*P* value
	CC (N = 105)	CA+AA (N = 36)		
**Clinical Stage**				
Stage I/II	75 (71.4%)	18 (50.0%)	1.00	*P* = 0.019 *
Stage III/IV	30 (28.6%)	18 (50.0%)	2.500 (1.148–5.446)	
**Tumor size** †				
≦ T2	74 (70.5%)	18 (50.0%)	1.00	*P* = 0.026 *
> T2	31 (29.5%)	18 (50.0%)	2.387 (1.098–5.188)	
**Lymph node metastasis**				
No	103 (98.1%)	33 (91.7%)	1.00	*P* = 0.072
Yes	2 (1.9%)	3 (8.3%)	4.682 (0.750–29.234)	
**Distant metastasis**				
No	101 (96.2%)	33 (91.7%)	1.00	*P* = 0.281
Yes	4 (3.8%)	3 (8.3%)	2.295 (0.488–10.790)	
**Vascular invasion**				
No	88 (83.8%)	29 (80.6%)	1.00	*P* = 0.654
Yes	17 (16.2%)	7 (19.4%)	1.249 (0.471–3.313)	
**Child-Pugh grade**				
A	83 (79.0%)	25 (69.4%)	1.00	*P* = 0.240
B or C	22 (21.0%)	11 (30.6%)	1.660 (0.709–3.887)	
**HBsAg**				
Negative	63 (60.0%)	20 (55.6%)	1.00	*P* = 0.640
Positive	42 (40.0%)	16 (44.4%)	1.200 (0.559–2.578)	
**Anti-HCV**				
Negative	50 (47.6%)	22 (61.1%)	1.00	*P* = 0.162
Positive	55 (52.4%)	14 (38.9%)	0.579 (0.267–1.252)	
**Liver cirrhosis**				
Negative	19 (18.1%)	7 (19.4%)	1.00	*P* = 0.857
Positive	86 (81.9%)	29 (80.6%)	0.915 (0.349–2.399)	

The ORs with analyzed by their 95% CIs were estimated by logistic regression models. Child-Pugh grades indicate the severity of cirrhosis: A = 5–6 points, B = 7–9 points and C = 10–15 points.

^†^ >T2 indicated the multiple tumor more than 5 cm or tumor involving a major branch of the portal or hepatic vein(s).

* *P* value < 0.05 as statistically significant.

**Table 5:** Odds Ratio (OR) and 95% Confidence Interval (CI) of clinical status and *ADAMTS14* rs61573157 genotypic frequencies in 141 HCC patients with tobacco consumption.

Variable	Genotypic frequencies		OR (95% CI)	*P* value
	CC (N = 119)	CT+TT (N = 22)		
**Clinical Stage**				
Stage I/II	83 (69.7%)	10 (45.5%)	1.00	*P* = 0.027 *
Stage III/IV	36 (30.3%)	12 (54.5%)	2.767 (1.096–6.483)	
**Tumor size** †				
≦ T2	82 (68.9%)	10 (45.5%)	1.00	*P* = 0.034 *
> T2	37 (31.1%)	12 (54.5%)	2.659 (1.055–6.704)	
**Lymph node metastasis**				
No	116 (97.5%)	20 (90.9%)	1.00	*P* = 0.126
Yes	3 (2.5%)	2 (9.1%)	3.867 (0.607–24.617)	
**Distant metastasis**				
No	114 (95.8%)	20 (90.9%)	1.00	*P* = 0.332
Yes	5 (4.2%)	2 (9.1%)	2.280 (0.413–12.572)	
**Vascular invasion**				
No	101 (84.9%)	16 (72.7%)	1.00	*P* = 0.164
Yes	18 (15.1%)	6 (27.3%)	2.104 (0.726–6.097)	
**Child-Pugh grade**				
A	93 (78.2%)	15 (68.2%)	1.00	*P* = 0.310
B or C	26 (21.8%)	7 (31.8%)	1.669 (0.616–4.524)	
**HBsAg**				
Negative	72 (60.5%)	11 (50.0%)	1.00	*P* = 0.358
Positive	47 (39.5%)	11 (50.0%)	1.532 (0.615–3.817)	
**Anti-HCV**				
Negative	58 (48.7%)	14 (63.6%)	1.00	*P* = 0.199
Positive	61 (51.3%)	8 (36.4%)	0.543 (0.212–1.391)	
**Liver cirrhosis**				
Negative	22 (18.5%)	4 (18.2%)	1.00	*P* = 0.973
Positive	97 (81.5%)	18 (81.8%)	1.021 (0.314–3.315)	

The ORs with analyzed by their 95% CIs were estimated by logistic regression models. Child-Pugh grades indicate the severity of cirrhosis: A = 5–6 points, B = 7–9 points and C = 10–15 points.

^†^ >T2 indicated the multiple tumor more than 5 cm or tumor involving a major branch of the portal or hepatic vein(s).

* *P* value < 0.05 as statistically significant.

### In silico characteristic nsSNPs on structural features of human ADAMTS14

**Fig 1:**
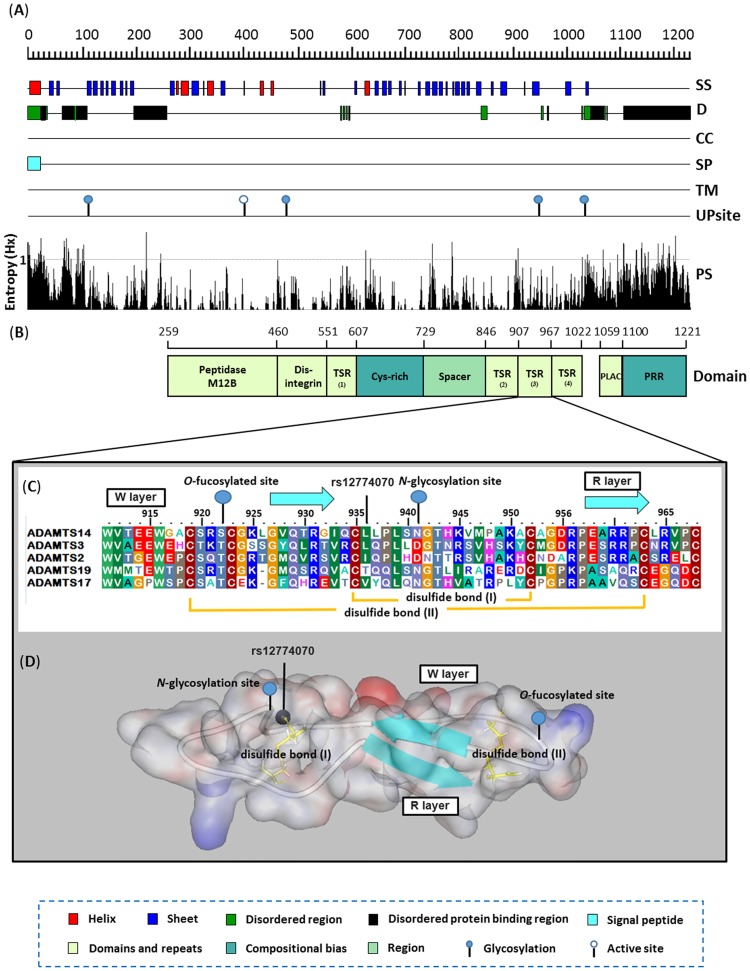
Proteome annotation diagram of *ADAMTS14* nsSNP rs12774070 by *in silico* approaches. **(A)** The *ADAMTS14* protein is first evaluated by sequence-based annotated methods with secondary structure (PSIPRED [SS]), disordered region (DISOPRED [D]), coiled region (COILS [CC]), signal peptides (SignalP [SP]) and transmembrane helices (TMHMM [TM]). In additional, active sites and glycoprotein sites are retrieved from UniProtKB database [UP sites]. **(B)** Indicating the level of conservation of residues by PSI-BLAST for each position using BioEdit Entropy algorithm [PS]. **(C)** Schematic representation of the full-length human *ADMDTS14* protein, domain symbols are drawn approximately to scale. Amino acids are colored according to residue type: blue, positive; red, negative; light blue, small; green, hydrophobic; light green, aromatic; brown, cysteine and gray, polar. The rectangles represent the key domain structures, Peptidase M12B (IPR013273), Disintegrin (IPR018358), Cys-rich (IPR006586), Spacer (IPR010294), PLAC (IPR010909), Pro-rich (IPR026086) and four TSR (IPR000884) processed by InterPro database. **(D)** The residues are conserved throughout five procollagen aminopropeptidase subfamily of ADAMTS proteases, including ADAMTS2 (NP_055059.2), ADAMTS3 (NP_055058.2), ADAMTS14 (NP_631894.2), ADAMTS17 (NP_620688.2) and ADAMTS19 (NP_598377.4), as shown by alignment of the protein sequences with Clustal Omega software. Numbering is for human *ADAMTS14*. The two tryptophans (Trp911 and Trp916) and two arginines (Arg960 and Arg961) that form W layers and R layers, respectively. Protein surface diagram depicts the homology model of 3^rd^ TSR domain of *ADAMTS14*. The ribbon indicates the Cα carbon of the 3^rd^ TSR domain characterized in this study. The blue ribbon, green sphere, red spheres and yellow sticks indicate the β-strands structure, rs12774070, potential *N*-linked, *O*-linked glycosylation sites and disulfide bonds, respectively.

Based on current computational protein annotation methods that predict general features a 22-amino acid signal peptide at the hydrophobic N-terminus of *ADAMTS14* is activated in the trans-Golgi network and leads to secretion, while no other obvious transmembrane and coil-coils regions of high hydropathy were present according to TMHMM [36] and COILS [37] predictions (Fig 1A). Otherwise, the basic *ADAMTS14* structure comprises a characteristic peptidase M12B catalytic domain and a carboxyl-terminal ancillary domain containing a disintegrin-like module, Cys-rich domain, Spacer, four TSRs and a unique sequence including PLAC (protease and lacunin) [38] that is embedded in a PRR domain (Fig 1B).

Fig 1C shows the amino acid position 941 of *ADAMTS14* (the position of the amino acid substitution corresponding to rs12774070). The 3^rd^ TSR domain is characterized by potential post-translational *O*-linked and *N*-linked glycosylation sites [39] with the sequences Cxx(S/T)CG and Nx(S/T) (residues 919–924 and 941–943; where C is cysteine, S is serine, T is threonine, N is asparagine and x is any amino acid) in a 20-residue stretch, respectively (Fig 1C). The ancillary domain is probably independent of the biological function and is more involved with the ECM association and the localization of the protease and its interaction partners for substrate specificity. Considering the essential role of *ADAMTS14* in various models of disease, we performed *in silico* profiling of evolution-based and homology three-dimensional molecular model of the 3^rd^ TSR domain (amino acid residues 911–968) of human *ADAMTS14* (Fig 1D). Based on the structural data provided, site-specific information such as for variant rs12774070 near the essential *N*-glycosylation site within its 3^rd^ TSR domain that can be regulated by its substrate recognition and cellular localization and which seems to involve a reduced affinity of *ADAMTS14* associated with ECM components can be appreciated (Fig 1D).

**Fig 2:**
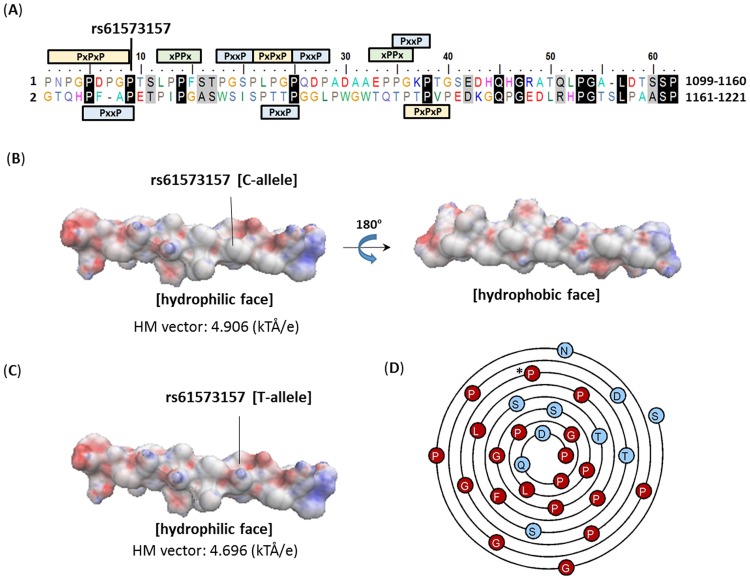
Amphipathic helix structure of a duplication of a 60-amino acid proline-rich domain at C-terminal *ADAMTS14*. **(A)** Alignment of a 60-amino acid duplication proline-rich repeats of the amphipathic helix (residues 1099–1160 and 1161–1121) present in with Clustal Omega software. The lines about the repeat 1 and below the repeat 2 show the three types of consensus linear motifs (PxPxP, PxxP and xPPx, where P is proline and x is any amino acid), respectively. The shading show the conserved residues and numbering is for the two repeats. Amino acids are colored according to residue type: blue, positive; red, negative; light blue, small; green, hydrophobic; light green, aromatic; brown, cysteine; and gray, polar. **(B)** C-allele and **(C)** T-allele of polymorphic *ADAMTS14* nsSNP rs61573157 regular α-helical conformation of Proline-rich domain. Electrostatic potential in the solvent-accessible surface representations colored code where red and blue represent the net negative and positive charges, respectively. The two structure through the axis of rotation 180 degree to across the opposite of helices. White color represents the total neutral positions. 3D hydrophobic moment vectors were calculated *in silico* in http://www.ibg.kit.edu/HM/. **(D)** Wenxiang diagram represents that characterizes the disposition of hydrophobic (red-filled circles) and hydrophilic (blue-filled circles) residues in α-helices. The black star symbol indicated the nsSNP rs61573157.

We further analyzed another affected variant (rs61573157), which has a unique PRR relative to the other ADAMTS family members, formed by a 122 amino acid stretch after the PLAC domain that is 25.4% proline and which contains a duplication of a 60-amino acid PRR with an amphipathic helix structure (Fig 2A). Searches of the human genome sequences reveal that at least four different mammalian proteins contain a homologous, similar PRR domain including *TPRX1*, *ARHGAP26*, *DMRTC2* and *COLEC12* (Figure B in S1 File).

To understand how the rs61573157 variant could affect the PRR structure, we constructed a modelling structure that was obtained from high resolution NMR data using 3D hydrophobic moment (HM) vector tool to characterize the surface polarity of the PRR domain. The helix-formed rods have amphipathic properties leading to the formation of a hydrophobic and a hydrophilic face with electrostatic surface (Fig 2B). Variant rs61573157 (P1108S) maps on to the hydrophilic face of the amphipathic helix of the PRR domain and is thought to constitute a potential binding site for ECM components. In addition, the liver-cancer associated risk [T]-allele creates the average HM vector of the *ADAMTS14* PRR domain compared to the [C]-allele, which results in a decreased length of HM vector [HM vector change: 4.5%, from 4.696 (kTÅ/e) to 4.906 (kTÅ/e)] (Fig 2B and 2C). Additionally, the proline residue is a very unusual amino acid, because the side-chain is cyclized back on to the backbone amide position that is extended with a helix conformation as the adaptor role. Wenxiang diagrams show the amphipathic characteristics and the residue distribution with most hydrophobic residues in one-half of the molecule and most hydrophilic residues in the other half (Fig 2D). These data suggested that the risk-associated variant rs61573157 located in the ancillary domain probably alters the specificity of their substrate-binding preferences and enzyme localization via ECM association and may affect the clinical characteristics of tumor stage and size in HCC patients with smoking habits (Tables 4 and 5).

## Discussion

Several studies suggested that chromosome 10q21 involved multiple genetic events have been implicated in the progression of multiple cancers [40–42]. Recent studies have reported that ADAMTS can exhibit both oncogenic and tumor-protective effects on tumor angiogenesis, an indispensable process vital for cancer progression and metastasis [43, 44]. Possible anti-angiogenic mechanism mediated by the TSR domains involved the cleavage of large thrombospondin 1 (TSP1) and TSP2 by *ADAMTS1* after being released from the ECM and are an established anti-angiogenic activity, suggestion that the TSR domain in proteinase is important for their anti-angiogenic function [45]. Therefore, we hypothesize that genetic variants of *ADAMTS14* may influence clinical outcomes in localized HCC patients with smoking behaviors. Our data reveal progressive clinical stage and increased tumor size among HCC smoking patients with at least one polymorphic A-allele of rs12774070 compared with those with homozygous C/C in case-control study. Our findings point to the role of variant rs12774070 in the TSR domain near the glycosylation site that may affect the TSR domain and can alter the cell-surface association and sequential processing mechanisms of *ADAMTS14*. We presented additional evidence in HCC, as elevated *ADAMTS14* gene expression was associated with more aggressive cancers and poorer clinical outcomes.

Furthermore, rs61573157 located in the PRR domain of the C-terminal ancillary domain of *ADAMTS14* was clinically examined probably because a consensus PxPxP motif had high binding capacity to E1A binding protein p300; the p300-p53 assembly reaction typically influences the alternative *TP53* acetylation [46]. Although directly testing this hypothesis was beyond the scope of the current study, evidence suggests that a flexible p300-binding motif exists within the PRR domain and is associated with the susceptibility to HCC in patients with smoking habits, suggesting that *ADAMTS14* down-regulation is associated with increased susceptibility to HCC. Hence, the present analyses increased our understanding of naturally occurring *ADAMTS14* variants, a lesion category that, although not infrequent, has been relatively neglected in terms of exploring the underlying pathogenic mechanisms. An improved understanding of these variants is a prerequisite for developing therapeutic approaches that can eventually ameliorate the clinical phenotype in patients harboring the corresponding lesions.

Smoking yields toxins with cytotoxic potential which induce the processes of hepatic inflammation and fibrosis. In addition, progressive fibrosis and cirrhosis should be closely monitored in patients with a high risk of developing HCC. Although biomarkers are not widely accepted as important clinical tools [47], they contribute valuable information for the management of patients with HCC with regard to surveillance, diagnosis, evaluation of treatment efficacy, and prediction of outcomes HCC is usually diagnosed in cirrhotic patients (60%-80%). Proteolytic processing occurs in the ECM during the resolution of fibrosis and might predispose to future injury. The non-invasive markers may further improve the prediction to evaluate liver fibrosis status and impaired liver regeneration.

In conclusion, this study comprises a comprehensive effort to supplement medical information regarding HCC pathogenesis and conducting additional bioinformatics analyses of a high number of patients to provide comprehensive evidence of the roles of *ADAMTS14* polymorphisms in HCC. Our results suggest that the *ADAMTS14* polymorphic variants in the *ADAMTS14* ancillary domains contribute to the occurrence and susceptibility to HCC in smoking patients. The joint effect of *ADAMTS14* polymorphism in the TSR domain and PRR domain markedly facilitated HCC development. Overall, our analyses provide deeper insights into naturally occurring genetic variants. Characterizing the molecular basis of mutations in cancer cells provides insight into tumorigenesis and accurate biomarkers on such types of variant are required for developing optimal therapeutic approaches that can eventually ameliorate the clinical phenotype in patients harboring the corresponding lesions.

## Supporting information

S1 File **Table A. Association of ADAMTS14 genotypic frequencies with HCC laboratory status. Figure A. ADAMTS14 gene structure and Haploview LD display analysis**. (A) ADAMTS14 is large gene and span 90K bp (from Chr.10 72,432,559 to 72,522,197; GRCh37.p.13). There are total 22 exons in this gene (NM_080722.3), the four selected SNPs, rs10823607, rs12774070, rs4747096 and rs61573157, are located on the exons 12, 19, 21 and 22, respectively. In the LD structure of SNPs-pairwise correlation coefficients in East Asian population (HCB+JPT) are shown (B) generated by Haploview version 4.2. A total of 34 haplotype blocks from 716 SNPs in the ADAMTS14 gene region were determined using the define blocks method “Four Gamete Rule” and LD colour scheme “Standard (D’/LOD)”. The four selected SNPs are shown as alleles of that region with recombination rate plotted and haplotypes display, (C) rs10823607 (529th), (D) rs12774070 (621th), (E) rs4747096 (669th) and (F) rs61573157 (689th), located on the LD map of haplotype blocks 22, 26, 31 and 32, respectively. **Figure B. Alignment comparison of amino acids sequence**. Alignment comparison of amino acids sequence of the amphipathic helix (residues 1098–1125) determined from five proline-rich region in proteins including TPRX1 (NP_940881.2), ARHGAP26 (NP_055886.1), DMRTC2 (NP_001035373.1) and COLEC12 (NP_569057.1) with Crustal Omega software. Amino acids are coloured according to residue type: blue, positive; red, negative; light blue, small; green, hydrophobic; light green, aromatic; brown, cysteine and grey, polar. Numbering is for human *ADAMTS14*.
(PDF)
