# Supplementary material for: Effects of ADAMTS14 genetic polymorphism and cigarette smoking on the clinicopathologic development of hepatocellular carcinoma
Source: PLoS One. 2017 Feb 23;12(2):e0172506. doi: 10.1371/journal.pone.0172506 (PMC5322915; doi:10.1371/journal.pone.0172506)
Supplement: S1 File — Table A. Association of ADAMTS14 genotypic frequencies with HCC laboratory status. Figure A. ADAMTS14 gene structure and Haploview LD display analysis. (A) ADAMTS14 is large gene and span 90K bp (from Chr.10 72,432,559 to 72,522,197; GRCh37.p.13). There are total 22 exons in this gene (NM_080722.3), the four selected SNPs, rs10823607, rs12774070, rs4747096 and rs61573157, are located on the exons 12, 19, 21 and 22, respectively. In the LD structure of SNPs-pairwise correlation coefficients in East Asian population (HCB+JPT) are shown (B) generated by Haploview version 4.2. A total of 34 haplotype blocks from 716 SNPs in the ADAMTS14 gene region were determined using the define blocks method “Four Gamete Rule” and LD colour scheme “Standard (D’/LOD)”. The four selected SNPs are shown as alleles of that region with recombination rate plotted and haplotypes display, (C) rs10823607 (529th), (D) rs12774070 (621th), (E) rs4747096 (669th) and (F) rs61573157 (689th), located on the LD map of haplotype blocks 22, 26, 31 and 32, respectively. Figure B. Alignment comparison of amino acids sequence. Alignment comparison of amino acids sequence of the amphipathic helix (residues 1098–1125) determined from five proline-rich region in proteins including TPRX1 (NP_940881.2), ARHGAP26 (NP_055886.1), DMRTC2 (NP_001035373.1) and COLEC12 (NP_569057.1) with Crustal Omega software. Amino acids are coloured according to residue type: blue, positive; red, negative; light blue, small; green, hydrophobic; light green, aromatic; brown, cysteine and grey, polar. Numbering is for human ADAMTS14. (PDF) [file pone.0172506.s001.pdf]

Table A. Association of *ADAMTS14* genotypic frequencies with HCC laboratory status.

Characteristic	α-Fetoprotein† (ng/mL)	AST† (IU/L)	ALT† (IU/L)	AST/ALT† ratio
**rs10823607**				
CC	2628.1 ± 816.0	140.4 ± 18.1	117.9 ± 14.1	1.51 ± 0.09
CT+TT	5194.9 ± 2712.3	92.1 ± 16.2	82.8 ± 15.8	1.39 ± 0.12
p value	0.258	0.274	0.312	0.618
**rs12774070**				
CC	3238.2 ± 1025.1	117.3 ± 13.1	106.7 ± 13.0	1.51 ± 0.10
CA+AA	2349.5 ± 1017.6	178.6 ± 46.9	129.9 ± 29.3	1.42 ± 0.10
p value	0.627	0.085	0.406	0.619
**rs4747096**				
AA	2786.5 ± 1173.9	120.3 ± 22.5	101.3 ± 12.1	1.35 ± 0.07
AG+GG	3157.1 ± 1088.8	142.3 ± 21.4	120.6 ± 19.0	1.59 ± 0.12
p value	0.821	0.489	0.440	0.152
**rs61573157**				
CC	2956.4 ± 917.0	126.8 ± 15.5	106.4 ± 11.8	1.51 ± 0.09
CT+TT	3244.6 ± 1502.5	165.1 ± 52.7	143.6 ± 42.8	1.39 ± 0.09
p value	0.893	0.357	0.255	0.568

Mann-Whitney U test was used between two groups.

† Mean ± S.E.

Figure A

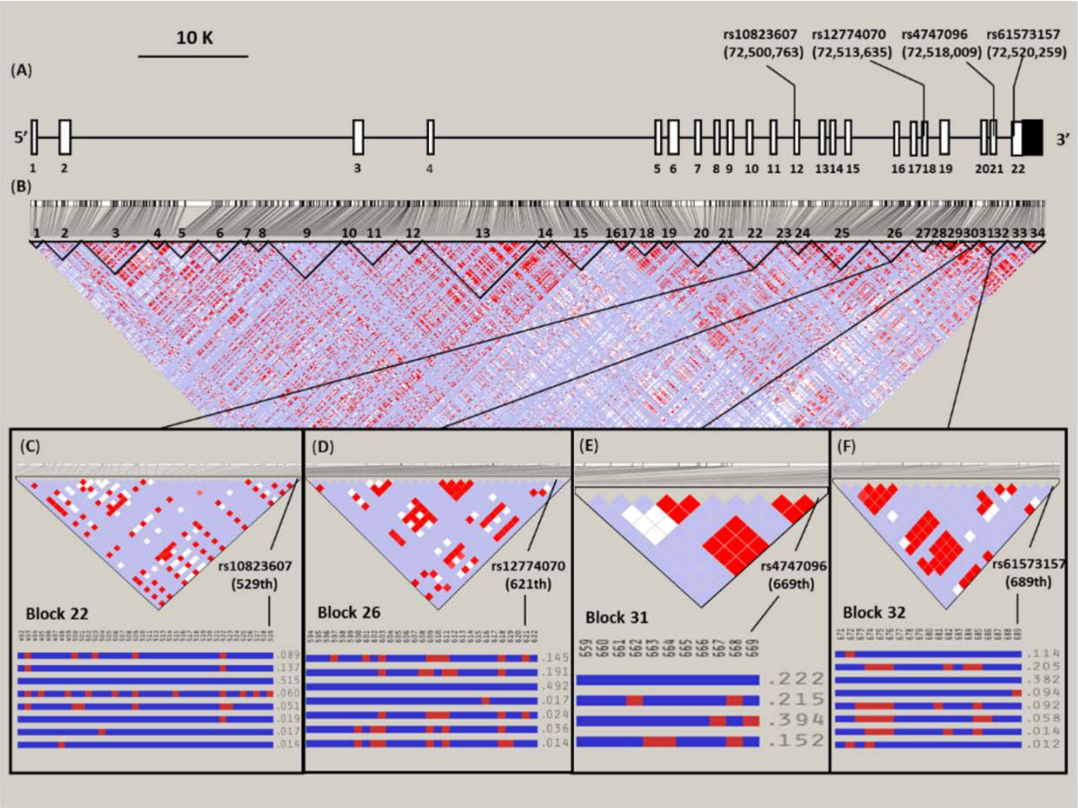

Figure A. ***ADAMTS14*** **gene structure and Haploview LD display analysis. (A)** *ADAMTS14* is large gene and span 90K bp (from Chr.10 72,432,559 to 72,522,197; GRCh37.p.13). There are total 22 exons in this gene (NM_080722.3), the four selected SNPs, rs10823607, rs12774070, rs4747096 and rs61573157, are located on the exons 12, 19, 21 and 22, respectively. In the LD structure of SNPs-pairwise correlation coefficients in East Asian population (HCB+JPT) are shown **(B)** generated by Haploview version 4.2. A total of 34 haplotype blocks from 716 SNPs in the *ADAMTS14* gene region were determined using the define blocks method “Four Gamete Rule” and LD colour scheme “Standard (D’/LOD)”. The four selected SNPs are shown as alleles of that region with recombination rate plotted and haplotypes display, **(C)** rs10823607 (529th), **(D)** rs12774070 (621th), **(E)** rs4747096 (669th) and **(F)** rs61573157 (689th), located on the LD map of haplotype blocks 22, 26, 31 and 32, respectively.

Figure B

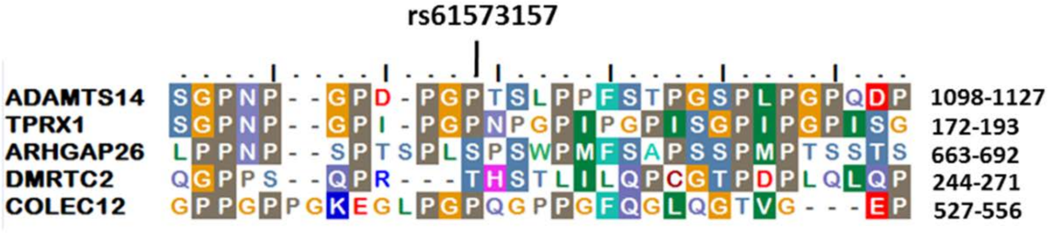

Figure B Alignment comparison of amino acids sequence of the amphipathic helix (residues 1098-1125) determined from five proline-rich region in proteins including TPRX1 (NP_940881.2), ARHGAP26 (NP_055886.1), DMRTC2 (NP_001035373.1) and COLEC12 (NP_569057.1) with Crustal Omega software. Amino acids are coloured according to residue type: blue, positive; red, negative; light blue, small; green, hydrophobic; light green, aromatic; brown, cysteine and grey, polar. Numbering is for human *ADAMTS14*.
